# Prevalence of Ticks Infesting Dairy Cattle and the Pathogens They Harbour in Smallholder Farms in Peri-Urban Areas of Nairobi, Kenya

**DOI:** 10.1155/2021/9501648

**Published:** 2021-12-10

**Authors:** Shepelo Getrude Peter, Hellen Wambui Kariuki, Gabriel Oluga Aboge, Daniel Waweru Gakuya, Ndichu Maingi, Charles Matiku Mulei

**Affiliations:** ^1^Department of Clinical Studies, Faculty of Veterinary Medicine, University of Nairobi, P.O. Box 29053-00625, Nairobi, Kenya; ^2^Department of Microbiology, Faculty of Health Sciences, University of Nairobi, P.O. Box 19676-00202, Nairobi, Kenya; ^3^Department of Public Health Pharmacology and Toxicology, Faculty of Veterinary Medicine, University of Nairobi, P.O. Box 29053-00625, Nairobi, Kenya; ^4^Department of Veterinary Pathology, Microbiology and Parasitology, Faculty of Veterinary Medicine, University of Nairobi, P.O. Box 29053-00625, Nairobi, Kenya

## Abstract

This study aimed at determining the tick species infesting dairy cattle in Nairobi, Kenya, and the pathogens they harbour. While ticks are well-known vectors of major bacterial pathogens of both veterinary importance and public health importance, few studies have investigated the range of the tick species and the associated pathogens, especially present in unique dairy production systems, which compromise animal welfare, such as those in peri-urban areas. A cross-sectional study was undertaken involving 314 randomly selected dairy cattle in 109 smallholder farms. Each animal was examined for attached ticks followed by morphological tick identification at the species level. Genomic DNA was extracted from each of the ticks, and 16S rDNA gene was amplified for pathogen identification. Sequencing of the amplicons and subsequent BLASTn analysis, multiple sequence alignment, and phylogenetic reconstruction were performed to confirm the species of the pathogens. Sixty-six (21.0%) of the cattle examined had ticks. A total of 94 adult ticks were found on the cattle, and of these, 63 (67.0%), 18 (19.1%), and 13 (13.8%) were in the genera *Rhipicephalus, Amblyomma,* and *Hyalomma,* respectively. Twelve tick species in *Rhipicephalus* genus and two in *Amblyomma* and *Hyalomma* genera were identified. Although *Rh. decoloratus* was the most prevalent tick (24.5% (23/94)), the emerging *Rh. microplus* (6.4% (6/94)) was also identified. The DNA of *Rickettsia* was detected in the ticks, with *Rickettsia conorii* in *H. rufipes* and *A. variegatum*, and *Rickettsia aeschlimannii* in *Rh. microplus* and *H. rufipes,* while *Ehrlichia ruminantium* and *E. canis* were in *A. variegatum.* In conclusion, the study reported a wide range of tick species present in the study area including *Rhipicephalus microplus,* which is an emerging tick species in parts of Kenya. The ticks harboured DNA of *Rickettsia* and *Ehrlichia*, highlighting possible animal and human health concerns. Hence, effective tick control strategies remain paramount to prevent potential diseases associated with the harboured pathogens.

## 1. Introduction

Ticks are important vectors for diseases of both veterinary and public health concerns. They are only second to mosquitoes in the transmission of animal and human pathogens [1]. They are known to transmit various pathogens including bacteria, rickettsia, protozoa, and viruses that are economically important to livestock [2] and those of zoonotic potential [3, 4]. In addition to the transmission of pathogens, they can cause tick worry and toxic reactions to animals [5]. The occurrence of the tick-borne diseases is related to the spatial distribution of the respective tick vectors [6]. The inevitable climate change, increase in human population, and constantly evolving changes in land-use patterns and livestock husbandry practices in Kenya are some of the reasons that have led to variations in the epidemiology and diversity of tick-borne diseases [7, 8]. There are limited studies in Kenya that have identified the tick species present and the pathogens they harbour. Such studies have mainly focused on pastoral areas [9, 10] and livestock-wildlife interfaces [11, 12] with hardly any focus on ticks present in unique production systems such as those in peri-urban areas of Nairobi.

Dairy cattle rearing is a common practice in the peri-urban areas of Nairobi County, Kenya, where they have been established to meet the ever-increasing milk consumption by the city's population of nearly 4.3 million people [13]. These production systems are characterized by substandard animal husbandry practices, which compromise animal welfare [14, 15]. The substandard animal husbandry practices are mainly a consequence of extensive land subdivision in peri-urban areas of the city resulting in small land sizes for cattle housing and minimal spaces for growing fodder [16]. Compromised animal welfare has been associated with decreased immunity, thereby predisposing animals to diseases [17]. Due to the land subdivision, the grasses and fodder for cattle in these production systems are sourced from various regions of the country with the risk of introducing a wide range of tick species to the animals.

A recent report by Peter et al. [18] indicated the infection of cattle with various tick-borne pathogens in these peri-urban areas of Nairobi. This study was carried out to further identify ticks found attached to those cattle and screen them for *Anaplasma, Ehrlichia,* and *Rickettsia* pathogens. Understanding the tick species in a given region has been used as a guide in preempting disease conditions to expect [19]. This information is therefore important in guiding tick control programs, hence preventing outbreaks of associated diseases.

## 2. Materials and Methods

### 2.1. Ethical Approval

This study was approved by the Biosecurity, Animal Use and Ethics Committee (BAUEC) of the Faculty of Veterinary Medicine, University of Nairobi, Kenya (FVM BAUEC/2016/122). Appropriate physical restrain of the cattle in a crush was done during the collection of the ticks to ensure that the animal did not struggle and all other animal welfare concerns were keenly adhered to. After a detailed explanation of the study to the animal owners, verbal consent was sort before recruiting the animals to the study.

### 2.2. Study Area

The study was carried out in peri-urban areas of Nairobi City County as previously described [20], and the method description partially reproduces the wording. Nairobi City, which is the capital of Kenya, is situated in this county. The county lies at 1.28333 latitude and 36.81667 longitude and 1795 m above sea level and consists of 17 subcounties, the highest number of administrative units in a single county in Kenya. For purposes of data collection, the county was mapped into four quadrants taking the central business district (CBD) as the center. In each of the quadrants, the subcounty with the highest cattle population was purposively selected. Therefore, the subcounties identified were Westlands (north quadrant), Kasarani (east quadrant), Lang'ata (south quadrant), and Dagoretti (west quadrant).

### 2.3. Study Design

This was a cross-sectional study conducted between January and May 2017 where dairy cattle recruited for a previous study [18] were examined for ticks on their various body parts. Within the subcounties, the databases kept by the subcounty veterinary officers were used as the sampling frames for random selection of farms in the study. Random sampling was used at the farm level to identify cattle that were included in the study. A total of 314 cattle in 109 dairy farms were recruited in the study. Each animal was restrained in a crush and physically examined for the presence of ticks on the head, ears, neck, dewlap, flanks, ventral abdomen, and perineal areas.

### 2.4. Tick Collection and Morphological Identification

All ticks found attached to the cattle were picked using forceps taking precautions not to break the mouthparts. They were stored in labelled containers containing 70% alcohol before transportation to the Parasitology Laboratory in the Department of Veterinary Pathology, Microbiology and Parasitology, University of Nairobi. Morphological identification of ticks was done to the species level based on taxonomic keys such as colour, shape, and size of capitulum, eyes, presence or absence of festoons, punctuation, groove on the scutum or conscutum, and presence or absence of adanal shield as described by Walker et al. [21] using a binocular microscope. Each identified tick was stored in a separate labelled collection tube containing 70% alcohol.

### 2.5. DNA Extraction from Ticks

The ticks were removed from the 70% alcohol, air-dried, and rinsed twice in distilled water before being dried on a filter paper. Genomic DNA was extracted from ticks using the DNeasy Blood and Tissue Kit (Qiagen, Hilden, Germany) following the manufacturer's instructions. DNA extraction was undertaken only on unengorged ticks to avoid PCR inhibition from excess erythrocytes as recommended by Silaghi et al. [22] and avoid confusion with DNA from the tick blood meal. The quality of the DNA was verified using QIAxpert slides in the QIAxpert machine (Qiagen, Hilden, Germany), and the integrity was confirmed by running 5 *μ*l of the eluted DNA using 1% agarose gel (Sigma, USA). The DNA was stored at −20°C awaiting analysis.

### 2.6. Polymerase Chain Reaction (PCR) Amplification and Sequencing of Pathogens' DNA in the Ticks

Polymerase chain reaction was performed on 16S rDNA gene using previously described primers and protocol [18]. For *Anaplasma* species, the forward primer ANAF 5′-TAGTGGCAGACGGGTGAGTA-3′ and a reverse primer ANAR 5′-AATTCCGAACAACGCTTGCC-3′ were used to yield an approximately 424 bp band, while for *Ehrlichia* species a forward primer EHRF 5′-AGCTGGTCTGAGAGGACGAT-3′ and a reverse primer EHRR 5′-GAGTGCCCAGCATTACCTGT-3′ targeting an approximately 838 bp of the 16S rDNA were used. Double-distilled water was used as the negative control. The PCR amplification reactions were performed in a thermal cycler (Applied Biosystems Veriti 96-Well Thermal Cycler, Thermo Fisher). The amplified products were electrophoresed using 1.5% agarose gel in Tris-borate-EDTA (TBE) buffer, pH 8, stained with ethidium bromide, and visualized using UV illuminator (UVP GelMax^®^ 125 Imager, USA). The sizes of the amplicons were determined using molecular ladder (GelPilot 1kb Plus Ladder (100), Qiagen, Germany). The resulting PCR amplicons were purified and Sanger sequenced at Macrogen Europe Laboratories (Amsterdam, the Netherlands) using the same forward and reverse primers as for the PCRs. The obtained sequences were viewed and manually verified using chromatogram peaks, edited, and assembled using CLC Main Workbench 6.8.3 Software (CLC Bio, Qiagen GmbH, Germany).

### 2.7. DNA Sequence Analysis

The sequences obtained from the 16S rDNA gene were analysed using Basic Local Alignment Search Tool Nucleotide (BLASTn), multiple sequence alignment (MSA), and phylogenetic reconstruction. BLASTn was used to confirm the sequence identities, while MSA and phylogenetic reconstruction identified the relationship between the study isolates and those from other regions of the world. Multiple sequence alignment was done using Log-Expectation (MUSCLE) v3.8.31 [23]. Sequence similarity was calculated using Clustal Omega to obtain identity matrix [24]. A phylogenetic reconstruction was done using MEGA 6.0 [25]. The evolutionary history was inferred using the maximum-likelihood method based on the Tamura–Nei model [26]. Initial trees for the heuristic search were obtained automatically by applying the neighbor-joining and BioNJ algorithms to a matrix of pairwise distances estimated using the maximum composite likelihood (MCL) approach and then selecting the topology with superior log-likelihood value. All positions containing gaps and missing data were eliminated. The percentage of replicate trees in which the associated taxa clustered together in the bootstrap test (1000 replicates) was shown next to the branches [27].

## 3. Results

### 3.1. Morphological Identification of Ticks

Of the 314 animals examined, 21.0% (66/314) were found to be infested with one or more ticks. A total of 118 adult ticks were found attached to the cattle at the time of sampling, but 24 were engorged; therefore, only 94 ticks were analysed further. Among the ticks analysed, twelve tick species in the three genera *Rhipicephalus* (*Rh.*), *Amblyomma* (*A.*), and *Hyalomma* (*H.*) were identified (Table 1). The majority (67% (63/94)) of them were *Rhipicephalus* species, while *Hyalomma* species were the least (13.8% (13/94)). In *Rhipicephalus* genera, *Rh.* (*b*)*. decoloratus* was the most prevalent tick species and only one tick was identified as *Rh. praetextatus*. *Rhipicephalus (boophilus) microplus* was also identified in 6% (6/94) of the examined ticks. *Amblyomma variegatum* and *Hyalomma rufipes* were the most prevalent in their respective genera. All the tick species were only found in cattle from Kasarani Subcounty.

### 3.2. Pathogen DNA Detected from Ticks Infesting Dairy Cattle in Smallholder Farms in Peri-urban Areas of Nairobi

Using primers previously used by Peter et al. [18] to detect *Anaplasma* species, 25.6% (24/94) of the samples yielded the expected PCR bands at approximately 424 bp. Eight representative amplicons were selected for sequencing. On BLASTn analysis, seven of them had DNA of *Rickettsia* and one *Anaplasma ovis* (Table 2).

The DNA of *Anaplasma ovis* was detected in *Rh. evertsi evertsi* tick, while all the three tick genera were found to harbour DNA of *Rickettsia* species. The DNA of *Rickettsia aeschlimannii* was detected in *Rh. (boophilus) microplus* and *Hyalomma rufipes* ticks. On the other hand, the DNA of *Ri. conorii* was detected in *Amblyomma gemma* and *A. variegatum* (Table 2). *Rickettsia conorii* was detected in 62.5% (5/8) of the sequenced amplicons, indicating sequence similarity of between 98.30% and 99.38%. *Rickettsia aeschlimannii* was confirmed in two of the sequenced amplicons (25% (2/8)) with a sequence similarity of between 99.30% and 99.76% (Table 2).

On analysis using primers for the detection of *Ehrlichia* species, of the 94 ticks that were analysed, 8.5% (8/94) were positive yielding PCR bands at approximately 838 bp. Five representative samples were sequenced for the detection of the pathogens. Three of the sequences, one from *Rh.* (*boophilus*) *decoloratus* and two from *A. variegatum,* were similar to *Ehrlichia canis* with a sequence identity of 99.76 to 100% (Table 3). One isolate from *A. variegatum* was 100% similar to *E. ruminantium,* while the other sequence from *Rh. sanguineus* was an unidentified *Ehrlichia* closely related to *Ehrlichia* spp. isolate KX987325 from Wuhan, China. Two *Amblyomma variegatum* ticks were observed to be coinfected with two pathogen DNAs each: one with *Ri. conorii* and *E. canis* (isolate 508B) and the other with *Ri. conorii* and *E. ruminantium* (isolate 524A) (Tables 2 and 3).

### 3.3. Multiple Sequence Alignments of the *Rickettsia* and *Ehrlichia* Species DNA Isolated from Ticks Infesting Dairy Cattle from Peri-Urban Areas of Nairobi

**Figure 1:**
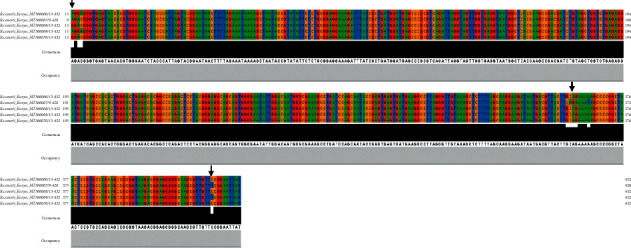
Multiple sequence alignment for Ri. conorii nucleotide sequences obtained from ticks infesting cattle in Nairobi, Kenya. The black arrows show regions of multiple nucleotide sequence polymorphisms (SNPs).

Multiple sequence alignment was done on the *Ri. conorii* and *E. canis* isolates detected from cattle in peri-urban areas of Nairobi, Kenya. The nucleotide sequences of *Rickettsia conorii* appeared genetically diverse with multiple nucleotide sequence polymorphisms (SNPs) (Figure 1) and nucleotide diversity of up to 4% (Table 4). In contrast, the nucleotide sequences for *Ehrlichia canis* were highly conserved.

### 3.4. Phylogenetic Positioning of the *Rickettsia* and *Ehrlichia* Species DNA Detected in Ticks Infesting Dairy Cattle from Peri-Urban Areas of Nairobi

**Figure 2:**
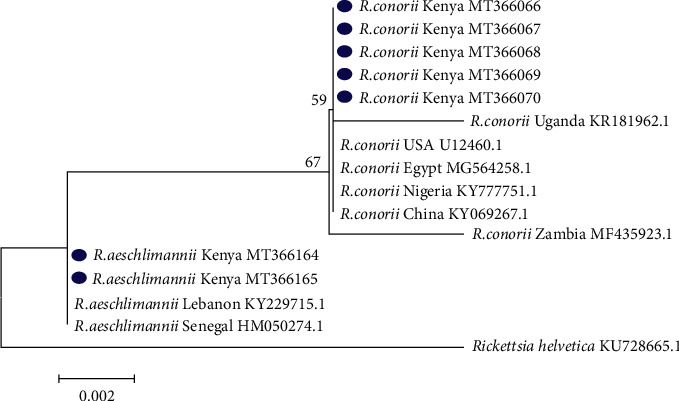
Maximum-likelihood tree of *Rickettsia* spp. reconstructed based on partial sequences of 16S rDNA gene with 1000 bootstrap replicates. The analysis involved 16 nucleotide sequences with seven from this study and nine others obtained from the GenBank. The tree indicates the phylogenetic relatedness of *Rickettsia* isolates obtained from ticks infesting cattle in peri-urban areas of Nairobi, Kenya, marked with blue dot and sequences from other countries. Rickettsia helvetica was used as an out-group. Sequence accession numbers are given at the end of each isolate.

**Figure 3:**
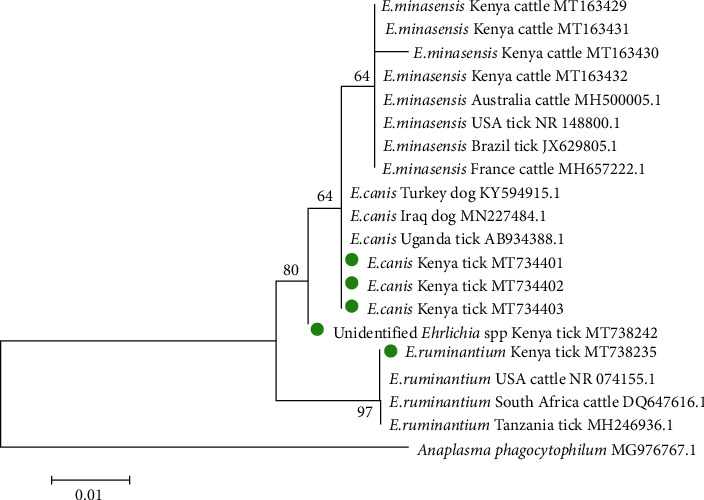
Maximum-likelihood tree of Ehrlichia spp. reconstructed based on partial sequences of 16S rDNA gene with 1000 bootstrap replicates. The tree is drawn to scale, with branch lengths measured in the number of substitutions per site. The analysis involved 20 nucleotide sequences with five tick isolates (green dots) from this study and the other fifteen obtained from the GenBank. The tree indicates the phylogenetic relatedness of Ehrlichia isolates from ticks infesting cattle in peri-urban areas of Nairobi, Kenya, marked with green dots and sequences from other countries. Anaplasma phagocytophilum was used as an out-group. Sequence accession numbers are indicated at the end of each isolate.

Phylogenetic analysis was done to confirm the DNA sequences of *Rickettsia* and *Ehrlichia* detected and identify the genetic relatedness to other isolates worldwide (Figures 2 and 3). *Ri. conorii* Kenyan isolates were closely related to those from the USA, China, Nigeria, and Egypt but differed from those from Zambia and Uganda. *Rickettsia aeschlimannii* Kenyan isolates were clustered together with an isolate from Senegal (KY229715.1) and Lebanon (HM050274.1) (Figure 2).

On the other hand, *Ehrlichia canis* isolates from the Kenyan ticks were closely related to dog isolates from Iraq and Turkey and a tick isolate from Uganda (Figure 3). *E. ruminantium* isolates from *A. variegatum* in this study were clustered together with a tick isolate from Tanzania and other isolates from cattle in South Africa and the USA. The unidentified *Ehrlichia* species was closely related to *E. canis* and *E. minasensis* but distantly from *E. ruminantium*.

### 3.5. Nucleotide Sequence Accession Numbers for the Pathogens Detected in the Identified Kenyan Ticks

The partial 16S rDNA gene sequences obtained from pathogens in ticks from this study were deposited in the GenBank under the following accession numbers: MT366066 to MT366070 for *Ri. conorii*, MT366164 to MT366165 for *Ri. aeschlimannii*, MT366207 for *Anaplasma ovis*, MT734401 to MT734403 for *E. canis*, MT738235 for *E. ruminantium,* and MT738242 for the unidentified *Ehrlichia* species.

## 4. Discussion

Tick infestation was relatively low in the study cattle with all the ticks collected being from Kasarani Subcounty. The problem of tick infestation and consequently tick-borne diseases has been well documented as a challenge in smallholder dairy farming in peri-urban areas of Nairobi [28] and especially in Kasarani Subcounty [29]. However, the increased use of acaricide, which is a common practice in smallholder dairy farms in Kenya [30] and the dry season at the time of sampling [21], may explain the low tick infestation reported in this study.

The high tick infestation in Kasarani Subcounty has been associated with open grazing lands that attract pastoral livestock from the neighboring Kajiado County, which are often heavily infested with ticks [31]. Despite the heavy infestation, there is usually minimal impact on the health of pastoral cattle since they exhibit a certain level of resistance to tick-borne diseases [30]. However, they contaminate the grazing pastures with the ticks they carry. Since cut and carry of fodder from the roadsides is the common method of feeding livestock in this area [16, 32], the ticks are simultaneously carried to the zero-grazed cattle.

Ticks in the genera *Rhipicephalus* including subgenera *boophilus*, *Amblyomma,* and *Hyalomma* were identified in this study. Ticks in these three genera have been previously reported in Kenya [33], Tanzania [34], Uganda [35], and Ethiopia [36]. The wide range of species identified in this study may have been influenced by the conducive climatic conditions that support tick vectors [4, 7]. Ticks in these genera consist of important species that transmit diseases of great economic impact to livestock production as well those of concern to human health [21].

The African blue tick (*Rhipicephalus* (*boophilus*) *decoloratus*) was the most prevalent tick species identified in the study cattle. This tick is the most widespread one host tick in Eastern, Central, and Southern Africa [37, 38]; therefore, these results were not surprising. It is known to transmit *Babesia bigemina* [39] and *Anaplasma marginale* [40], which are endemic pathogens in the study area [28]. Indeed, *A. marginale* was detected in the sampled cattle from where the ticks were collected [18].

The Asian blue tick (*Rh.* (*boophilus*) *microplus*) was also identified in the study cattle. This tick was first reported in the coastal areas of Kenya by Hoogstraal and Walker [41], and since then, no reports of this tick have been made until recently when Kanduma et al. [12] characterized this tick in Kwale County using molecular markers. These tick species are highly invasive and have been reported to replace *Rh.* (*boophilus*) *decoloratus* in areas where both tick species exist [42, 43]. They are most economically important in the subgenera *boophilus* due to their role in the transmission of fatal *Babesia bovis* infection [44] and the novel *E. minasensis* [45]. The identification of this tick species in other parts of the country implies the possible emergence of infections in areas that had no previous reports. In fact, Adjou Moumouni et al. [46] and Peter et al. [18] reported the presence of *B. bovis* and *E. minasensis* for the first time in cattle in peri-urban areas of Nairobi, respectively, suggesting the possible presence of the vector tick *Rh. boophilus microplus* as reported in this study. Uncontrolled animal movement in Kenya [7] and continued climate change have been suggested to be the key drivers in the spread of different tick species [47].

In this study, the brown dog tick (*Rhipicephalus sanguineus*), which typically infests dogs, was found infesting the cattle. The majority of the farms where the cattle were sampled were observed to keep dogs, possibly explaining the presence of these ticks. This may result in infection of cattle with dog-related pathogens such as *Anaplasma platys* as observed previously in the study cattle [18] and reported in other parts of the world [48, 49]. *Rhipicephalus sanguineus* was also found to harbour unidentified *Ehrlichia* species, which clustered in a different clade from *E. canis,* which is the pathogen that *Rh. sanguineus* is known to vector [50, 51]. Whole-genome sequencing of this unknown *Ehrlichia* pathogen would shed light on its identity, and studies on its pathogenicity on cattle and the role of *Rh. sanguineus* in its transmission are needed.

*Anaplasma ovis*, the causative agent of ovine anaplasmosis, was detected in one *Rhipicephalus evertsi evertsi* attached to cattle similar to the report by Berggoetz et al. [52]. Although this pathogen is commonly isolated in small ruminants [53], the sharing of hosts by the different tick species may enable ticks to acquire multiple pathogens from different blood meals [54]. The detection of these pathogens in the ticks does not always imply vector competence [6, 55], but mechanical transmission may occur when susceptible hosts such as cattle come in contact with infected ticks [54].

*Amblyomma variegatum* and *A. gemma* were identified in this study. *Amblyomma* species are among the most important tick species in Africa transmitting devastating animal diseases such as heartwater disease [43, 56]. These tick species have previously been identified and reported in Kenya [10, 33]. *Rickettsia conorii* was detected in *A. variegatum* ticks as previously reported from a study by Mutai et al. [38] in Kenya. Although *Ri. conorii* has been detected frequently in *Rh. sanguineus,* which is the documented vector tick [57, 58], it can also be detected in other tick species [59]. *Amblyomma* species have been known as the major reservoirs for the DNA of *Rickettsia* in Africa [60], and hence, it was not surprising to detect this pathogen in these ticks. The high attraction of *Amblyomma* ticks to humans [1] increases the risk of human infection by this pathogen.

In this study, *Amblyomma variegatum* was also found to harbour *E. canis* and *E. ruminantium* and in two cases coinfected with the DNA of *Rickettsia* spp. It was not surprising to detect *E. ruminantium* in *A. variegatum* ticks since they are the documented competent vectors for this pathogen [61, 62] and have previously been reported in Kenya [63]. Since *A. variegatum* has strongly been implicated in the transmission of *Rickettsia* species [64, 65], the detection of both *Rickettsia* species and *E. ruminantium* is possible [66]. The aggressive feeding habit by this tick species on various hosts [10] may explain the detection in the tick of *E. canis*, which is a pathogen of dog, and the coinfection reported in this study.

*Hyalomma rufipes* and *Hyalomma truncatum* were also identified in this study. These tick species have been reported previously in various parts of Kenya [33, 56, 63]. *Rickettsia aeschlimannii* was detected in *Hyalomma rufipes* ticks in this study. *Hyalomma* species are the documented vectors for *Ri. aeschlimannii* [60]. This pathogen was first detected and characterized in *Hyalomma marginatum* in Morocco [67], and since then, it has been isolated in different species of *Hyalomma* ticks in Kenya [9, 63], Ethiopia and Chad [68], Senegal [59], and Europe [69, 70]. Additionally, *Ri. aeschlimannii* was also detected in *Rh. microplus* tick. The detection of this pathogen in *Rh. (boophilus) microplus* is not surprising since Mutai et al. [38] and Reye et al. [54] detected this pathogen in its closely related species of *Rh. (boophilus) annulatus* attached to cattle from Kenya and Nigeria, respectively. Moreover, Pretorius and Birtles [71] also identified *Ri. aeschlimannii* in *Rh. appendiculatus* tick from South Africa. This indicates that *Ri. aeschlimannii* can be found in many other tick species although the vector competence for these tick species needs to be evaluated.

Although vector competence for some of the tick species found carrying pathogens identified in this study is yet to be established, their recognition in these ticks expands the knowledge on pathogens harboured by ticks infesting dairy cattle in peri-urban areas of Nairobi. The surveillance of pathogens in ticks has been viewed as a good start to preempt pathogens that could potentially infect animals and people [19]. Additionally, the identification of tick species and the detection of the pathogens they harbour in various areas of a country have been viewed as a prerequisite for developing appropriate tick control programs during targeted tick control [2].

In this study, *Ri. conorii* and *Ri. aeschlimannii,* which are zoonotic pathogens, were detected in ticks infesting cattle. *Rickettsia conorii* has been associated with febrile disease causing severe morbidity [72] and even fatality in tourists returning from Kenya [73], while *Ri. aeschlimannii,* which is a closely related pathogen, manifests with a mild disease [74]. Infection of humans with rickettsiosis occurs from contact with domestic animals or pets that act as reservoir host from which tick bites occur [54]. Indeed, Thiga et al. [75] reported a higher seroprevalence and titers of spotted fever group (SFG) rickettsiosis among pastoralists keeping large numbers of livestock than other communities in Kenya. The febrile disease from rickettsiosis has been commonly confused for malaria and typhoid, especially in Africa including Kenya where these diseases are endemic [76, 77]. In Kenya, there is evidence that rickettsiosis contributes to a great percentage of febrile cases reported in hospitals [78].

Since ticks remain infected with rickettsiosis for life while transmitting transstadially and transovarially [58], a public health risk is posed by their detection in ticks [54, 74]. Additionally, since dogs and ruminants are the implicated domestic reservoirs for this rickettsiosis [79], a country-wide surveillance to understand the status of the domestic reservoirs would be informative. Additionally, further characterization of the *Rickettsia* species and strains found in ticks and cattle using more specific primers targeting outer membrane proteins (Omp A and OmpB), citrate synthase, and 17 kDa proteins [80, 81] is necessary.

The 16S rDNA gene was used to infer phylogenetics of pathogens detected in the ticks from this study. *Rickettsia species* have a highly conserved genome, and their mitochondrial 16S rDNA has been previously used to detect tick microbiome [82–84]. *Rickettsia conorii* detected in this study showed high diversity and were closely related to an isolate from Nigeria, the USA, and China but differed from those from Uganda and Zambia. This indicates that different variants of the *Rickettsia* may exist in the study area with some that may be unique to Kenya. *E. canis* isolates were highly conserved and similar to those isolated from Turkey, Iraq, and neighboring Uganda, a suggestion that the strains may be similar to those isolated worldwide and their introduction may be associated with the international pet movement, which has been associated with pathogen spread [85].

The limitation of this study was that it was carried out during the dry season, therefore may not provide the whole spectrum of the tick species and the associated pathogens that would be expected in the study area. Future studies covering both the dry and wet seasons would provide a greater diversity of the ticks and their pathogens in these peri-urban areas.

## 5. Conclusion

Dairy cattle in peri-urban areas of Nairobi, Kenya, were infested with different tick species in the genera *Rhipicephalus*, *Amblyomma,* and *Hyalomma.* The ticks harboured DNA of various *Ehrlichia* species including the dog-associated *Ehrlichia canis* and zoonotic *Rickettsia* pathogens. This highlights the animal health threat and public health concern posed by the infestation of these ticks on the dairy cattle. Therefore, effective tick control remains paramount for the control of these pathogens. The role of cattle as alternative hosts for the dog-associated pathogens such as *E. canis* detected in ticks infesting cattle needs to be investigated, especially when cattle are in close proximity to the dogs. There is a need for molecular characterization of the identified tick species using specific genes such as interspacer (ITS) and CO1 genes to confirm the presence of the emerging invasive *Rh. microplus* ticks in various counties in Kenya.

## Figures and Tables

**Table 1:** Distribution of the tick species identified from dairy cattle in peri-urban areas of Nairobi, Kenya.

Tick species	No. of ticks collected (%, *n* = 94)
*Rhipicephalus* (Rh.) species	
*Rh. (boophilus) decoloratus*	23 (24.5)
*Rh. evertsi evertsi*	11 (11.7)
*Rh. pulchellus*	11 (11.7)
*Rh. (boophilus) microplus*	6 (6.4)
*Rh. sanguineous*	5 (5.3)
*Rh. simus*	3 (3.2)
*Rh. appendiculatus*	3 (3.2)
*Rh. praetextatus*	1 (1.2)
*Amblyomma* (A.) species	
*A. variegatum*	15 (15.6)
*A. gemma*	3 (3.2)
*Hyalomma* (H.) species	
*H. rufipes*	8 (8.5)
*H. truncatum*	5 (5.3)
Total	**94 (100)**

**Table 2:** Pathogens' DNA detected from ticks collected from dairy cattle in peri-urban areas of Nairobi, Kenya.

Isolate	Accession no. (this study)	Tick species	Accession no. of highest BLASTn match	Pathogen DNA detected	% identity
522A	MT366207	*Rh. evertsi evertsi*	MG869525.1	*Anaplasma ovis*	99.77
281C	MT366164	*Rh. (boophilus) microplus*	HM050274.1	*Ri. aeschlimannii*	99.76
290B	MT366165	*H. rufipes*	HM050274.1	*Ri. aeschlimannii*	99.30
286A	MT366066	*A. gemma*	MG564259.1	*Ri. conorii*	98.38
524A	MT366070	*A. variegatum*	MG564259.1	*Ri. conorii*	99.30
519A	MT366069	*A. variegatum*	MG564259.1	*Ri. conorii*	98.30
501A	MT366067	*A. variegatum*	MG564259.1	*Ri. conorii*	99.30
508B	MT366068	*A. variegatum*	MG564259.1	*Ri. conorii*	98.38

**Table 3:** *Ehrlichia* species DNA detected from different tick species collected from cattle in peri-urban areas of Nairobi, Kenya.

Isolate	Accession no. (this study)	Tick species	Accession no. of highest BLASTn match	*Ehrlichia* spp. DNA detected	% identity
396B	MT734401	*Rh. (boophilus) decoloratus*	KY594915.1	*E. canis*	100.0
508B	MT734402	*A. variegatum*	KX987326.1	*E. canis*	99.76
524A	MT734403	*A. variegatum*	KX180945.1	*E. canis*	100.0
524AR	MT738235	*A. variegatum*	NR_074155.1	*E. ruminantium*	100.0
277C	MT738242	*Rh. sanguineous*	KX987325.1	*Unidentified Ehrlichia spp.*	100.0

**Table 4:** Pairwise percent identity matches of 16S rDNA sequences of *Ri. conorii* isolated from ticks infesting dairy cattle in Nairobi, Kenya. The numbers denote the nucleotide identity rates found between the sequences.

Isolate	501A	286A	519A	508B	524A
501A	100.0	96.0	96.1	98.1	97.7
286A	96.0	100.0	99.8	97.0	96.8
519A	96.1	99.8	100.0	97.2	96.2
508B	98.1	97.0	97.2	100.0	99.8
524A	97.7	96.8	96.2	99.8	100.0

## Data Availability

The data used in this manuscript are available from the corresponding author on request. The DNA sequence of the pathogens analysed is available on GenBank using the accession numbers indicated in this manuscript.
